# Engineered trivalent immunogen adjuvanted with a STING agonist confers protection against *Trypanosoma cruzi* infection

**DOI:** 10.1038/s41541-017-0010-z

**Published:** 2017-04-10

**Authors:** Andrés Sanchez Alberti, Augusto E. Bivona, Natacha Cerny, Kai Schulze, Sebastian Weißmann, Thomas Ebensen, Celina Morales, Angel M. Padilla, Silvia I. Cazorla, Rick L. Tarleton, Carlos A. Guzmán, Emilio L. Malchiodi

**Affiliations:** 1 Universidad de Buenos Aires, Facultad de Farmacia y Bioquímica, Cátedra de Inmunología and Instituto de Estudios de la Inmunidad Humoral (IDEHU), UBA-CONICET, Buenos Aires, Argentina; 2 Universidad de Buenos Aires, Facultad de Medicina, Departamento de Microbiología, Parasitología e Inmunología and Instituto de Microbiología y Parasitología Médica (IMPaM), UBA-CONICET, Buenos Aires, Argentina; 3 Department of Vaccinology and Applied Microbiology, Helmholtz Center for Infection Research, Braunschweig, Germany; 4 Universidad de Buenos Aires, Facultad de Medicina, Departamento de Patología, Instituto de Fisiopatología Cardiovascular, Buenos Aires, Argentina; 5 Center for Tropical and Emerging Global Diseases, Athens, GA USA

## Abstract

The parasite *Trypanosoma cruzi* is the causative agent of Chagas disease, a potentially life-threatening infection that represents a major health problem in Latin America. Several characteristics of this protozoan contribute to the lack of an effective vaccine, among them: its silent invasion mechanism, *T. cruzi* antigen redundancy and immunodominance without protection. Taking into account these issues, we engineered Traspain, a chimeric antigen tailored to present a multivalent display of domains from key parasitic molecules, combined with stimulation of the STING pathway by c-di-AMP as a novel prophylactic strategy. This formulation proved to be effective for the priming of functional humoral responses and pathogen-specific CD8^+^ and CD4^+^ T cells, compatible with a Th1/Th17 bias. Interestingly, vaccine effectiveness assessed across the course of infection, showed a reduction in parasite load and chronic inflammation in different proof of concept assays. In conclusion, this approach represents a promising tool against parasitic chronic infections.

## Chagas disease: protecting from chronic parasitic disease

An amalgamation of parasitic proteins may be the first effective vaccine against the as yet untreatable chronic phase of Chagas disease. The infliction, caused by the parasite *Trypanosoma cruzi* (*T. cruzi*), is the world’s leading cause of infectious cardiac inflammation and puts one-sixth of the population of Latin America at risk of infection. International collaborators led by Emilio Malchiodi, of the University of Buenos Aires, Argentina, constructed a vaccine (dubbed ‘Traspain’) comprised of key *T. cruzi* proteins alongside a novel ‘adjuvant’—designed to promote the efficacy of a vaccine by activating inflammatory responses. The chimera and adjuvant combination elicited a promising immune response and also showed the capacity to prevent tissue damage caused by chronic infection. Multi-part vaccines such as Traspain offer an attractive direction for research into vaccines against chronic parasitic infections.

## Introduction

*Trypanosoma cruzi* is the etiological agent of Chagas disease, the world’s leading cause of infectious myocarditis. It is recognized by WHO as a neglected tropical disease in Latin America, where one-sixth of the population is at risk of contracting the infection.^1^

The infection is a complex zoonosis transmitted by several hematophagous Triatomine species. Despite the potent immune response the parasite triggers in the mammalian host, *T. cruzi* is able to persist, establishing a chronic infection. One hundred years following its discovery, there are still no effective vaccines or drugs to prevent or treat the chronic phase of the infection.

As *T. cruzi* spends most of its time in mammals as amastigote form replicating in the cytosol of host cells, cell-mediated immunity is essential for controlling the parasite.^2^ However, it has been shown that the cytotoxic T lymphocyte (CTL) response developed is restricted to a few epitopes of the Transialidase (TS) superfamily,^3^ giving rise to the question of immunodominance as an immune evasion mechanism.^4^ The redundancy of some *T. cruzi* antigens and the parasite silent invasion mechanism contribute to the lack of detection of infected cells during the first moment of entrance and represent a challenge in the design of anti-*T. cruzi* prophylactic vaccines. This scenario allows speculation about whether a chimeric immunogen could broaden the immune response triggered by vaccination and achieve better protection levels. To test this hypothesis, we engineered Traspain, a structure-based chimeric antigen, relying on several properties of three regions of *T. cruzi* proteins: (1) immunogenicity, (2) presence of reported and predicted major histocompatibility complex class I binding peptides, (3) protection capacity, (4) expression profile, and (5) structural signature. Thus, we selected the N-terminal domain of Cruzipain (Cz)—the major cistein protease—, the central region of amastigote surface protein 2 (ASP2)—an antigen expressed exclusively during the intracellular stage—, and an inactive transialidase (iTS)—a major antigen and virulence factor—, to generate a chimeric antigen presenting a multivalent display of key parasitic molecules.

Subunit vaccines need not only a good immunogen but also the right adjuvant. In search of novel components that can enhance cell-mediated immunity, we employed 3′5′-c-di-AMP (c-di-AMP), a cyclic di nucleotide (CDN), in vaccination protocols by the intranasal route. CDNs are STING agonists that activate IRF3, NF-kB, and STAT6, inducing type I IFN and pro-inflammatory cytokines;^5, 6^ originally described as bacterial second messengers associated with different metabolic process. Mammalian cells have also an eukaryotic counterpart, 2′5 ′-c-GAMP, as part of their DNA sensing machinery.^7^ These small molecules have been recently introduced as adjuvants.^8^ Here, we show how tailored antigen combined with this novel adjuvant represents a promising strategy for vaccines against parasitic infections.

## Results

### Construction, expression and characterization of Traspain

**Fig. 1:**
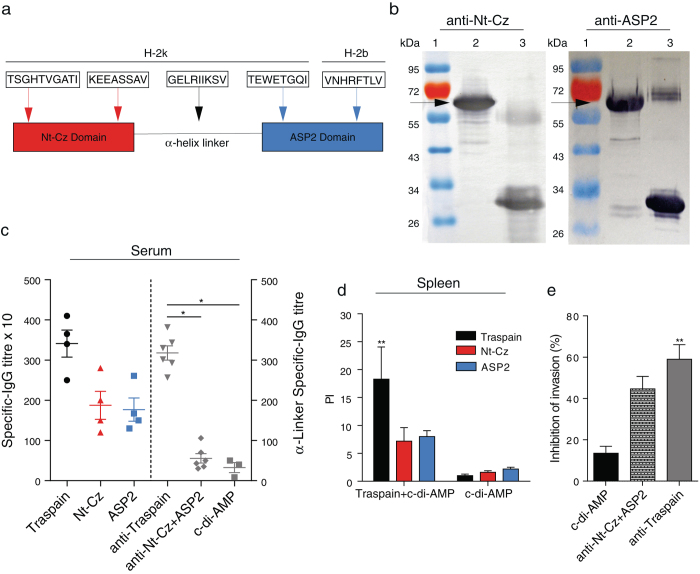
Characterization of Traspain as a chimeric immunogen. Schematic representation of Traspain. *Arrows* point at CD8^+^ T cell epitopes included in the design with its respective mouse MHC-I haplotype (**a**). Immunochemical identity by Western blot. Domain-specific polyclonal antibodies (pAb) were used as primary antibody. SDS-PAGE gels were loaded as follows, lines: 1-MWM, 2-Traspain, 3-Nt-Cz (*left*) or ASP2 (*right*), *arrows* point at Traspain band (**b**). Specific antibodies response. Titers were determined by ELISA in serum samples from mice vaccinated with either Traspain or antigen combination plus c-di-AMP at 15 days post vaccination. Plates were coated with Traspain, Nt-Cz, ASP2 (*left*) or GELRIIKSV *α*-linker-peptide (*right*). * *p* < 0.05, one-way ANOVA Kruskal-Wallis test + Dunn’s multiple comparisons test (**c**). Proliferative response in vaccinated mice 30 days after the last dose. Spleen cells were re-stimulated with 10 µg/ml of the indicated protein. Results are expressed as proliferation index (PI), *n* = 6 mice per group, **p < 0.01, 2-way-ANOVA + Tukey’s multiple comparisons test (**d**). Neutralization assay of pAb generated upon vaccination. *n* = 5–6 per group, **p < 0.01, one-way-ANOVA Kruskal-Wallis test + Dunn’s multiple comparisons test (**e**). Results are expressed as mean ± SEM and are representative of at least three independent experiments

Traspain was designed containing the Nt-Cz-domain, an *α*-helix linker from iTS and the central region of ASP2 (Fig. 1a). It was expressed in *E. coli* BL21 (DE3) and the immunochemical identity was determined by Western blot where Traspain was recognized by polyclonal antibodies specific for the main domains (Fig. 1b).

### Immunization with Traspain+c-di-AMP achieved an equal level of priming at both humoral and cellular levels

As Traspain antigen contains domains from different protein families of the parasite, we decided to test whether any interference was developed between the main regions of the construction when the protein was administered to C3H mice in the presence of a STING agonist as adjuvant.

Antibodies raised against Traspain were able to recognize Nt-Cz, ASP2 and GELRIIKSV peptide from the *α*-helix linker in an indirect ELISA-assay (Fig. 1c). Remarkab﻿ly, a similar level of Abs was detected against both Nt-Cz and ASP2, indicating absence of interference and inmunodominance at the humoral level. GELRIIKSV-specific IgG were detected in mice vaccinated with the chimeric molecule but not in controls (c-di-AMP) nor in Nt-Cz+ASP2 vaccinated mice that lack this motive﻿. The lower titer of this specificity was expected considering the 9-mer peptidic nature of the coating molecule.

Considering the report of ASP2 immunodominant T cell epitopes in natural infection,^9^ we analyzed the performance of Traspain+c-di-AMP to prime cell-mediated immune responses against each domain. No differences were detected in the proliferation levels of the Nt-Cz and ASP2-specific subpopulations, PI: 7.17 ± 2.44 and 8.02 ± 1.03, respectively; representing nearly half of the whole response (PI: 18.2 ± 5.78) upon restimulation with Traspain (Fig. 1d).

### Antibodies elicited by Traspain+c-di-AMP show neutralization capacity

*T. cruzi*-specific antibodies have been shown to be beneficial for the development of functional parasite-specific CD8^+^ T cells upon challenge.^10^ Traspain-specific-antibodies proved to be functional since incubation of trypomastigotes of Tulahuen strain with serum of vaccinated mice significantly decrease in vitro invasion of non-phagocytic and Raw-cells (Fig. 1e). Interestingly, the percentage of inhibition was slightly higher in Traspain+c-di-AMP group compare with sera from mice vaccinated with the main antigens combined.

### Immunization with Traspain+c-di-AMP primes a balanced immune response associated with a Th1/Th17 profile

**Fig. 2:**
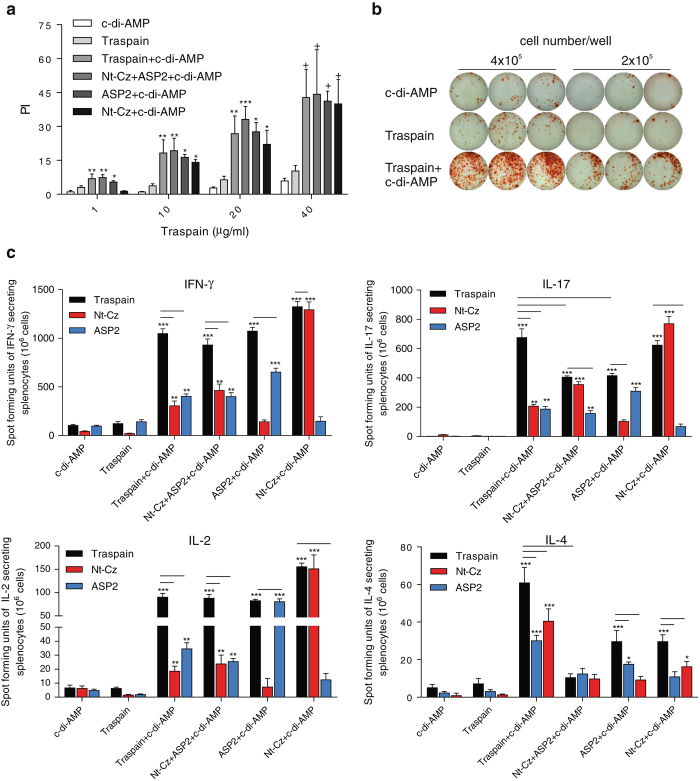
Cellular immune response in vaccinated mice. Proliferative dose-response curve of spleen cells harvested 30 dpi from vaccinated mice upon increasing concentrations of Traspain. Proliferation index (PI) (**a**). Secreted cytokines by ELISPOT assay at 30 dpi. Representative ELISPOT plate showing IFN-*γ* producing colonies of spleen cells from indicated groups cultivated in the presence of 10 µg/ml of Traspain (**b**). Pooled-splenocytes were re-stimulated with the indicated protein and mean number of spot forming units were determined for IFN-*γ*, IL-17, IL-2, or IL-4 (**c**). Results are expressed as mean ± SEM, *n* = 18 from 6 mice per﻿ group. One-way-ANOVA+Dunnett multiple comparison test. *p < 0.05, **p < 0.01, ***p < 0.001 comparing with c-di-AMP group. *Bars* indicate significant difference (*p* < 0.05) between the indi﻿cated category of stimulus and groups, two-way-ANOVA + Tukey’s multiple comparisons test. All results are representative of two independent experiments

Considering that the cellular immune response is essential to control intracellular pathogens like *T. cruzi*, we evaluated the ability of different vaccine candidates to elicit cell-mediated immunity. First, the proliferative potential was evaluated in vitro upon restimulation with Traspain. All vaccinated mice displayed a strong proliferative response against Traspain in a dose dependent manner (Fig. 2a).

The presence of Traspain, ASP2 and Nt-Cz-specific IFN-*γ*, IL-17, IL-2, and IL-4 secreting cells was assessed by ELISpot assay (Fig. 2b, c). Spleen cells from neither c-di-AMP nor Traspain groups secreted cytokines upon restimulation. However, all antigen+c-di-AMP-immunized groups secreted cytokines upon antigen reencounter with a profile compatible with a Th1/Th17 bias. Thus, splenocytes from mice immunized with Traspain+c-di-AMP predominantly secreted IFN-*γ* (11-fold increment over control), IL-2 (15-fold) and IL-17 (340-fold) upon *ex-vivo* restimulation with Traspain. In addition, we detected low levels of IL-4-specific secreting cells; being the ratio IFN-*γ*_(Traspain+c-di-AMP−c-di-AMP)_/IL-4_(Traspain+c-di-AMP−c-di-AMP)_ ≈ 17. Although the magnitude of IFN-γ and IL-2-secreting cells in Nt-Cz+ASP2+c-di-AMP group was similar to that of Traspain+c-di-AMP, the levels of IL-17 secreting cells were significantly less incremented. When we compared the groups that received antigens alone, an analogous scenario was observed for the ASP2+c-di-AMP group being the Nt-Cz+c-di-AMP group more similar in magnitude to Traspain+c-di-AMP.

The numbers of IL-2-Traspain-specific secreting cells were comparable in all antigen+c-di-AMP immunized groups a fact that correlates with the similar level of proliferation observe﻿d.

Furthermore, to confirm the absence of immunodominance towards the domains of the chimeric molecule, we also performed restimulation of spleen cells with the main antigens (Fig. 2c). Interestingly, Traspain immunized splenocytes respond to ASP2 and Nt-Cz in a similar fashion, revealing a balanced response to both of them.

### Antigen-specific CD4^+^ T cells upregulate CD154 and secrete IFN-*γ* and TNF-α

**Fig. 3:**
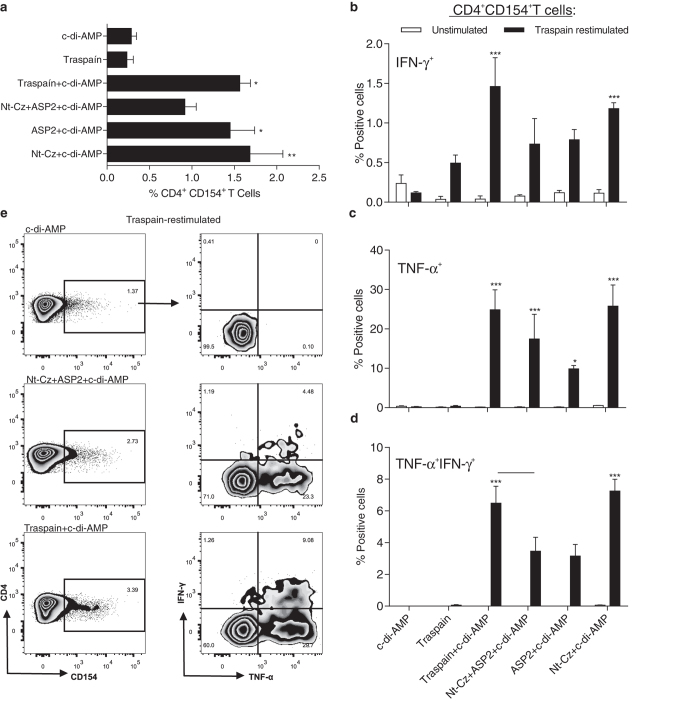
Antigen-specific CD4^+^ T cells generated during vaccination become activated and secrete cytokines upon re-encounter with the cognate antigen. Spleen cells from individual mice were harvested 30 days after last dose an﻿d﻿ transient expression of CD154^+^ (CD40L) was analyzed as an indicator of T cell activation upon antigen recall (**a**). Results are expressed as subtracted mean(restimulated mean—unstimulated mean) ± SEM. TNF-*α* and IFN-*γ* production by activated CD154^+^CD4^+^ T cells. Percentage of cytokine producing cells was determined for IFN-*γ* ^+^ (**b**), TNF-*α* ^+^ (**c**), or TNF-*α* ^+^IFN-*γ* ^+^ cells (**d**). Flow cytometry plots for the indicated groups showing the gating strategy (**e**). *p < 0.05, **p < 0.01, ***p < 0.001 comparing with c-di-AMP. One-way-ANOVA + Bonferroni multiple comparisons test (**a**) and two-way-ANOVA + Bonferroni multiple comparisons test (**b**–**d**), *bar* indicate *p* < 0.05 between groups, *n* = 4 per group. All results are representative of two independent experiments

In order to characterize the functionality of the CD3^+^CD4^+^ T cells, we performed FACS analysis of spleen cells from vaccinated and control mice. As shown in Fig. 3a, CD4^+^ T cells transiently upregulated CD154 upon restimulation with Traspain in all immunized mice. Moreover, most cells positive for this marker were also cytokine-producing cells compared with controls, as shown in Fig. 3b–e. In consistence with the results obtained in the ELISPOT assay, the group that received Nt-Cz+ASP2+c-di-AMP presented an immune response not as robust as Traspain+c-di-AMP, showing lower levels of activated CD154+ cells and nearly half of the percentage of triple positive CD4+CD154+TNF-α+IFN-γ+ T cells upon antigen recall. Activation of CD4 T cells was similar for the antigen alone+c-di-AMP groups although citoquine positive cells were higher in the Nt-Cz+c-di-AMP group after protein restimulation.

Altogether, these results highlight the ability of the formulation to generate high quality multifunctional CD4^+^ T cells that may help not only during the priming of CD8^+^ T cells, but also to activate infected macrophages during *T. cruzi* infection.

### Mucosal administration of Traspain+c-di-AMP is able to prime systemic parasite-specific CD8^+^ T cells

**Fig. 4:**
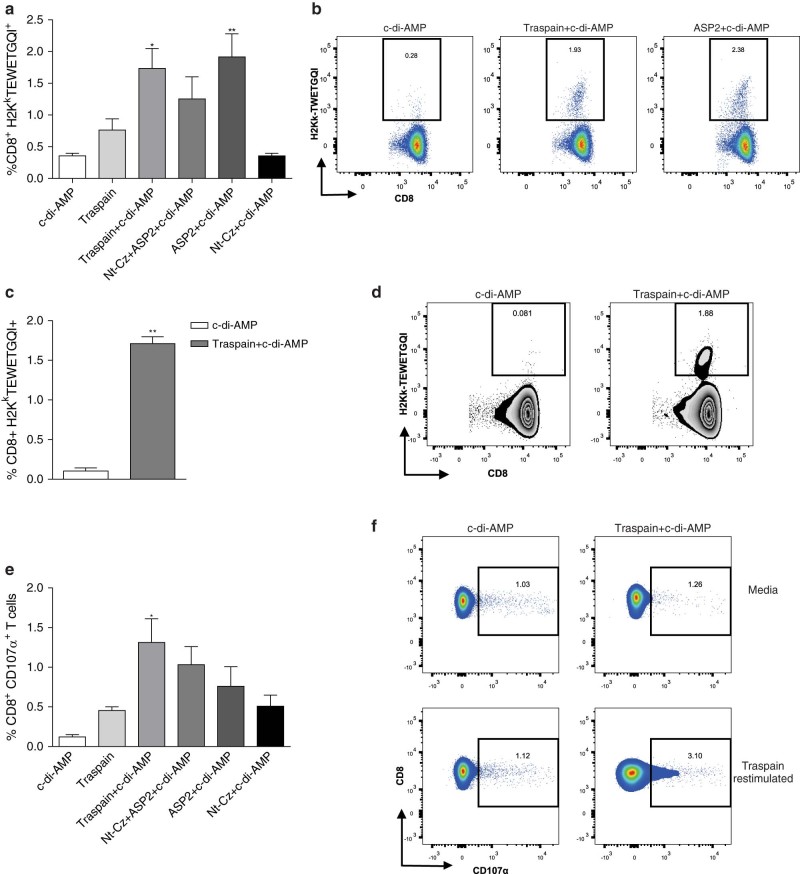
CD8^+^ T cell mediated immune responses of immunized mice. Priming of pathogen-specific CD8^+^ T cells by vaccination at 30 dpi (**a**). Representative dot-plots for the indicated groups (**b**). Levels of circulating pathogen-specific CD8^+^ T cells in blood for Traspain+c-di-AMP vaccinated and control mice (**c**) and representative zebra-plots of each group (**d**). Frequency of CD107α^+^ CD8^+^ T cells calculated upon antigen re-stimulation of spleen cells from each group (**d**). Representative dot-plots of the indicated groups (**e**). Results are expressed as mean (**a**, **c**) or subtracted mean (**e**) ± SEM and are representative of two independent experiments, *n* = 4 per group. **p < 0.01, *p < 0.05, one-way-ANOVA + Bonferroni multiple comparisons test (**a**, **e**). **p < 0.01 Mann–Whitney test (**c**)

Due to the key role of cytotoxic T cells in controlling parasite load during the acute phase of infection, we analyzed the ability of c-di-AMP to prime antigen-specific CD8^+^ T cells as a correlate of protection. Immunization with candidates plus c-di-AMP was able to prime parasite-specific CD8^+^ T cells for the immunodominant epitope of ASP2 as we determined by H2K^k^-TEWETGQI multimer staining albeit levels tend to be lower in the Nt-Cz+ASP2+c-di-AMP group. As expected, we did not detect an expansion of CD8+ T cells with this specificity in Nt-Cz+c-di-AMP vaccinated animals (Fig. 4a, b). Interestingly, ﻿these cells were also recirculating as we detected them in blood in the Traspain+c-di-AMP group (Fig. 4c, d).

Additionally, the functionality of CD8^+^ T cells was determined by its capacity to degranulate *ex-vivo* upon re-encounter with Traspain. We observed a higher frequency of CD8^+^ T cells expressing CD107α in immunized groups compared with the c-di-AMP control (Fig. 4e, f). Moreover, Traspain+c-di-AMP vaccinated mice showed the best cytotoxic potential as detected by the upregulation of this marker.

### Re-expansion capacity and functionality of vaccine-specific CD8^+^ T cell response upon *T. cruzi* infection

**Fig. 5:**
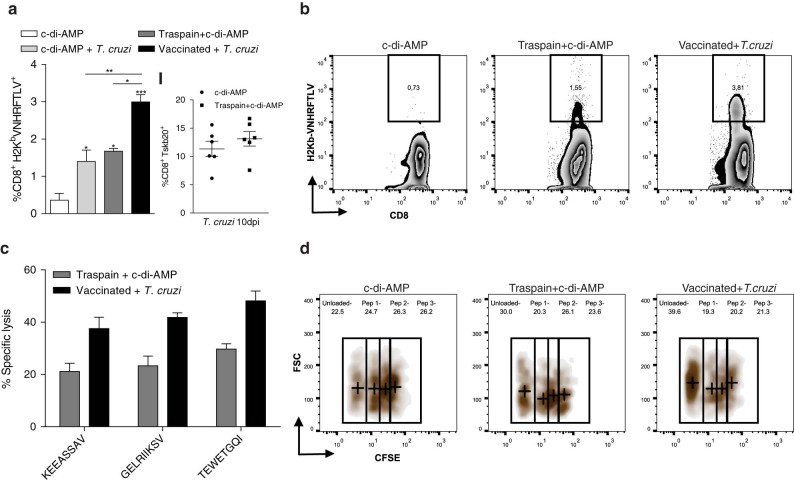
Pathogen exposure triggers an expansion of antigen-specific memory CD8^+^ T cell subset. Blood of C57BL/6 mice immunized (30 dpi) or immunized and challenged with 250,000 CL culture trypomastigotes (10 dpi) was analyzed for VNHRFTLV-specific CD8^+^ T cells by tetramer staining (**a**). Percentage of pathogen-specific, vaccine unrelated CD8^+^ T cells (Tskb20) by tetramer staining in blood (I). Zebra-plots showing VNHRFTLV-tetramer^+^ population on indicated groups (**b**). In vivo CTL assay (**c**). Spleen cells from C3H donor mice were loaded with peptides, KEEASSAV (Nt-Cz domain), GELRIIKSV (α-linker-motive), and TEWETGQI (ASP2 domain), or unloaded. Cells were stained with CFSE, mixed in an equal ratio, and i.v. injected to naïve (c-di-AMP), Traspain+c-di-AMP immunized or Traspain+c-di-AMP vaccinated and *T. cruzi* infected mice at 45 dpi (Vaccinated + *T. c﻿ruzi*). Representative density-plot showing percentage of CFSE populations in the indicated groups. *Crosses* represent the median value of each dimension in each gate (**d**). pep 1: KEEASSAV, pep 2: GELRIIKSV, pep 3: TEWETGQI. Results are expressed as mean ± SEM, and represent at least three independent experiments, *n* = 6 per group (**a**, **b**) and *n* = 3 per group (**c**, **d**), ***p < 0.001, **p < 0.01, *p < 0.05. 1way-ANOVA + Tukey’s multiple comparisons test (**a**)

With the aim of determining the impact of *T. cruzi* infection on pre-existing cell-mediated immunity, the percentage of H2K^b^-VNHRFTLV tetramer^+^ CD8^+^ T cells from vaccinated C57BL/6 mice was evaluated. Similar to what we found for the H2K^k^ haplotype, Traspain+c-di-AMP-vaccinated mice were able to prime pathogen-specific CD8^+^ T cells from the ASP2 region of the chimeric antigen on the H2K^b^ background. These cells were further expanded after *T. cruzi* CL infection, where we observed a two-fold increase in the frequency of H2K^b^ tetramer positive cells compared with both infected non-vaccinated and vaccinated not infected mice (Fig. 5a, b). In addition, the presence of antigen-specific CD8^+^ T cells did not affect the ability of the immune system to recognize and prime CD8^+^ cells against an unrelated parasite peptide as Tskb20 from TS (Fig. 5a I).

To compare functional aspects of the CTL response, we analyzed the lysis of peptide-pulsed splenocytes in an in vivo CTL assay in traspain-vaccinated C3H mice before and after *T. cruzi* challenge (Fig. 5c). In accordance with the CD107α staining, Traspain+c-di-AMP mice were able to lyse TEWETGQI-pulsed splenocytes. Notably, the vaccination approach was able to prime subdominant CTL targets from different regions of the construction as well (Nt-Cz domain, *α*-linker region) in a similar fashion. This fact highlights the lack of immunodominance of Traspain components and the efficacy of the formulation to increase the breadth of the CTL response in vaccinated mice.

### Traspain+c-di-AMP confers protection against different models of *T. cruzi* murine infection

**Fig. 6:**
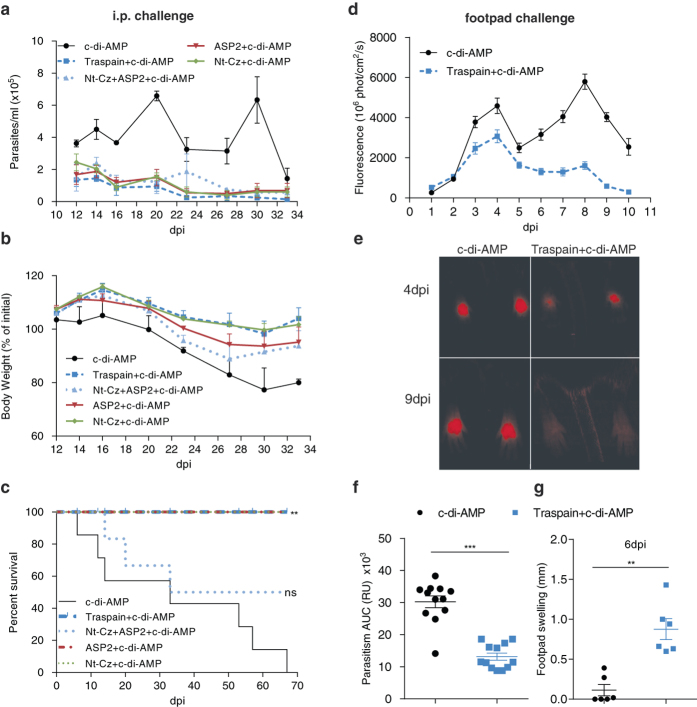
Vaccine efficacy during the acute phase of *T. cruzi* infection. Vaccinated C3H mice were challenged 30 days after the last dose with a lethal intraperitoneal (i.p.) dose of *T. cruzi* RA strain trypomastigotes and analyzed for: parasitemia (**a**), body weight loss (**b**) and survival (**c**), *n* = 6 per group, **p < 0.01, Log-Rank Test, ns: not significant vs. control. Footpad challenge of the indicated groups with trypomastigotes of the CL-tdTomato strain. Mean fluorescence intensity of feet determined by in-vivo imaging, between 1 and 10 dpi (**d**). Representative images from 4 to 9 dpi showing the control of parasite replication at the site of infection in vaccinated animals (**e**). Area under the curve of fluorescence per site of infection (*n* = 12) (**f**). Footpad swelling at 6 dpi (**g**). Footpad diameter was measured using a caliper. *Data* show the mean difference between day 0 and 6 per mouse. **P < 0.01, ***p < 0.001, Mann–Whitney test. The results are representative of at least three independent experiments

To analyze vaccine efficacy during the acute phase of infection, we vaccinated female C3H mice with candidates plus c-di-AMP and challenged them with highly virulent blood trypomastigotes of *T. cruzi* RA strain, a combination of parasite-mouse strain that proved suitable for this outcome of interest. All vaccinated mice were able to control parasitemia, remarkably the highest reductio﻿n (6-fold) was observed in Traspain vaccinated mice (mean area under the parasitemia curve ±SEM, c-di-AMP: 330±20, Traspain+c-di-AMP: 54±9, Nt-Cz+ASP2+c-di-AMP: 102±22, ASP2+c-di-AMP: 95±24, Nt-Cz+c-di-AMP: 91±12) (Fig. 6a). This fact also correlates with a decrease in the weight loss (Fig. 6b). A 18% weight reduction was observed in control mice after the first peak of parasitemia at 27dpi, whereas Nt-Cz+ASP2+c-di-AMP showed a 12% los﻿s being the immunized group with the worst performance. Similar protection levels were observed in Traspain, ASP2 or Nt-Cz+c-di-AMP groups in terms of survival rate (Fig. 6c). However, Nt-Cz+ASP2+c-di-AMP group failed to achieve equal protection. As a ﻿whol﻿e﻿, animals immunized with strategies different from Traspain+c-di-AMP presented an intermediate performance.

Since *T. cruzi* has many ways to evade the immune response and cause a chronic infection, it is essential to control parasite replication during the first moment of its entrance. To assess this matter, we determined the levels of parasitism at the site of infection by employing CL-strain trypomastigotes expressing the tandem dimeric protein, tdTomato. As virulence of these recombinant parasites is compromised, we employed male C57BL/6 mice considering its higher susceptibility to *T. cruzi* infection.^11–13^

As shown in Fig. 6d and e, the levels of fluorescence as a surrogate marker of parasite replication in situ were reduced in vaccinated mice in contrast with control animals. The early control of parasite replication was associated with an increase in the inflammation at the site of infection (Fig. 6f, g).

### Vaccine effectiveness for the prevention of tissue damage associated with chronic *T. cruzi* persistence

**Fig. 7:**
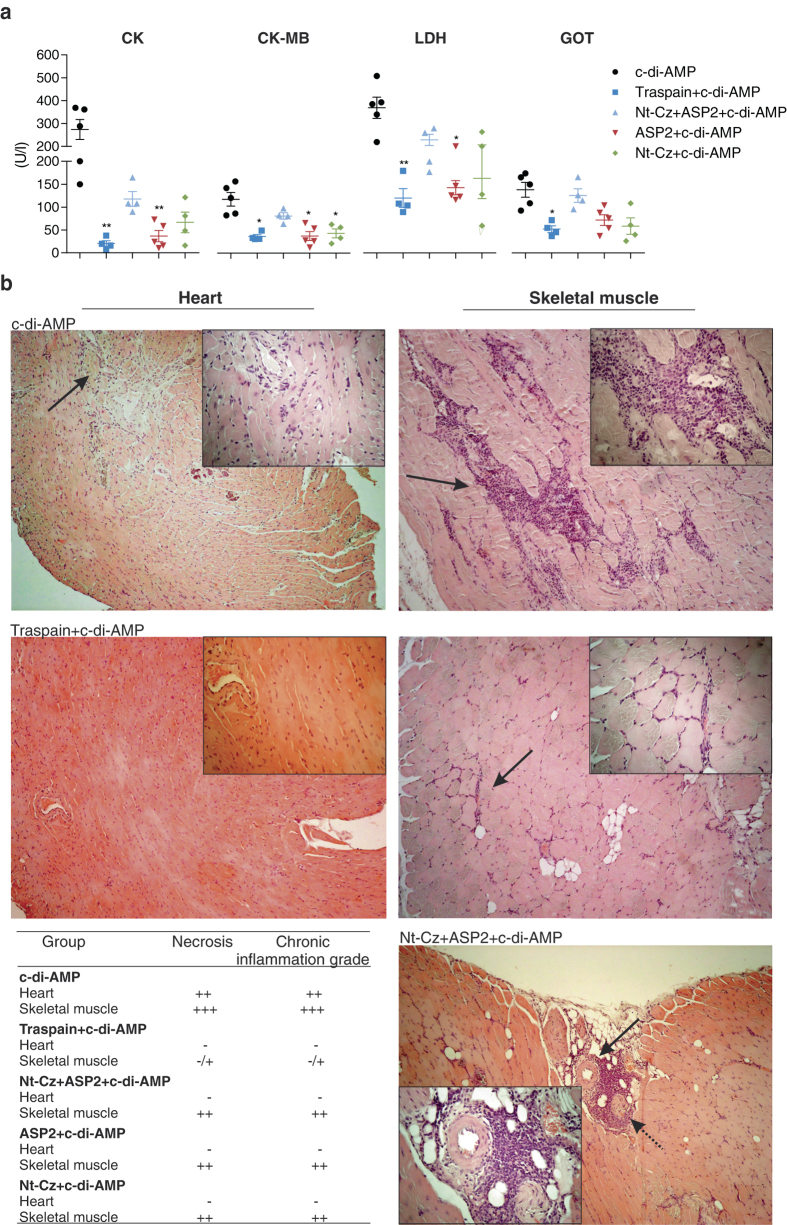
Vaccine effectiveness for the prevention of tissue damage**.** Serum activity of cardiomyopathy-associated enzymes from immunized and infected mice. Creatinine kinase (CK), creatinine kinase MB isoform (CK-MB), lactate dehydrogenase (LDH, U/I ×10), and glutamate oxaloacetate transaminase (GOT) (**a**). Results are expressed as mean ± SEM. *p < 0.05, **p < 0.01, one-way-ANOVA Kruskal-Wallis test + Dunn’s multiple comparisons test. Histopathological analysis of *T. cruzi*-target organs during the chronic phase of infection. Representative tissue sections (H&E stained) for the indicated groups (**b**). Magnification level: 100×. *Insets*: 400×. Table shows Inflammation score semi-quantitatively evaluated for each group (-, absence of inflammation; number of crosses indicate degree of inflammation). *Solid arrows* point to mononuclear cell infiltrates. *Dashed arrow* points to a nerve associated with mononuclear cell infiltrates. Results are representative of two independent experiments

The progression of *T. cruzi* infection can lead to parasite-induced damage in the target tissues. To assess whether vaccination approaches were able to diminish tissue injury, we determined the activity levels of serum enzymes associated with tissue damage. Vaccinated animals were able to control tissue injury during the chronic phase of the infection, showing less serum activity of CK and its cardiac isoform (CK-MB), as well as LDH and GOT compared to control-infected mice (Fig. 7a). To confirm this data, we analyzed the presence of necrosis and chronic inflammation in tissue sections from cardiac and skeletal muscle as endpoints. As Fig. 7b shows, tissue sections of control animals presented strong inflammatory infiltrates, and necrosis, which was always associated with the presence of mononuclear cells and higher levels of dystrophic calcification, all of which are consistent with a severe tissue damage scenario. Comparison between immunized groups revealed that all of them were able to control heart damage. However, only mice immunized with Traspain+c-di-AMP presented absence or a low level of skeletal muscle damage as well as mononuclear infiltrate in c﻿ontrast with the other groups, proving a benefit of this chimeric antigen vaccination protocol.

## Discussion

The rational design of Traspain as a trivalent inmunogen was based on a series of criteria that guided us to select each of the candidate to be incorporated in the chimeric gene. Thus, the Nt-Cz was chosen based on its protective capacity and excluding the C-terminal domain that distracts the immune response.^14^ It has a key role as a source of B and T cell epitopes. Secondly, the sequence from iTS was selected based on the *α*-helix structure that it adopts in the native conformation. In Traspain, it works as a linker between the N- and C-terminal domains acting as a molecular ruler, a fact that may contribute to the independent folding of each region.^15^ As we have shown ﻿it is also a source of subdominant CD8^+^ T epitopes. Finally, the ASP2 central region was incorporated based on its protective properties, its location within the parasite membrane and expression pattern.^16, 17^ It represents a potent target for the CTL response.^18, 19^ Noteworthy, the B cell immune response triggered by the chimeric antigen Traspain proved to be directed against both main domains in a similar fashion and in lesser extend, against a 9-mer peptide of the linker, as expected  (Fig. 1).

Similar to previous reports,^20^ the profile of the immune response triggered by c-di-AMP vaccination was associated with a Th1/Th17 bias and was detected, although not in the same magnitude, in all animals that received antigens+c-di-AMP. Thus, we observed a 300-fold induction of IL-17 secreting cells in the Traspain+c-di-AMP group compared with c-di-AMP alone and 200-fold for the group that received Nt-Cz+ASP2 combined. The strong production of this cytokine is associated on one hand to the adjuvant per se and on the other to the mucosal route of administration.^21^ IL-17 has been associated with protection during the acute phase of infection, contributing to parasite control and increasing survival of infected animals.^22^ Moreover, it has been described as a mechanism by which regulatory neutrophils are recruited to avoid the damage of an otherwise exaggerated Th1 response.^23^ Interestingly, it has been recently found that Th17 cells confer stronger protection than Th1 cells.^24^ This fact may partially explain the better control of *T. cruzi* infection of Traspain immunized mice compared to Nt-Cz+ASP2 vaccinated animals.

Flow cytometry data indicate that CD4^+^ T cells primed during vaccination were able to secrete cytokines and transiently express CD154 upon antigen recall *ex-vivo*. Interestingly, CD4^+^ T cells have an essential role in the efficient priming of CD8^+^ responses. The CD8^+^ T cells primed in the absence of T-cell-help are impaired in terms of their ability to respond to a secondary re-encounter with antigen, being compromised not only in the magnitude, but also in the quality of the CTL response. Upregulation of CD154 by CD4^+^ T cells plays a pivotal role during DC-licensing, a key step for the efficient priming of CD8^+^ T cells and optimal secondary CTL responses.^25, 26^ This fact is also important in the context of *T. cruzi* infection considering that helpless CD8^+^ T cells are of lower intensity and unable to control *T. cruzi* acute infection.^27^ In this scenario, it is reasonable to assume that vaccination with c-di-AMP could be able to prime pathogen-specific CD8^+^ T cells. Indeed, we detected CD8^+^ CTL in spleen and blood of Traspain+c-di-AMP vaccinated mice (Fig. 4a–d). Furthermore, these cells were able to degranulate upon antigen re-stimulation. Interestingly, they showed re-expansion capacity upon pathogen exposure, increasing not only in frequency (Fig. 5a) but also in functionality as determined by the ability to lyse peptide loaded target cells (Fig. 5c). Traspain vaccination was able to prime functional CD8 T cells directed against both dominant and subdominant (Nt-Cz and α-linker region) CTL epitopes. Altogether, these results highlight the ability of c-di-AMP to trigger cross-presentation of proteins as well as in vivo cross priming of specific CTL.

Moreover, a series of proof of concept studies were carried out challenging vaccinated animals with *T. cruzi* from different strains. Antigens+c-di-AMP were able to control acute infection in terms of parasitemia, weight loss, and survival (Fig. 6). Interestingly, during the chronic phase of the infection, vaccinated mice showed a decreased serum activity of tissue damage-associated enzymes. More importantly, Traspain+c-di-AMP vaccinated mice showed the lowest level of mononuclear infiltrate in skeletal muscle, compared with others groups (Fig. 7). These animals were also able to control parasite replication during the first days of infection, a fact that was associated with an earlier pro-inflammatory response at the site of infection. Further experiments are necessary to determine the etiology of the inflammatory process and the involvement of circulating or resident memory T cells.^28, 29^ As the outcome of interest for an anti-*T. cruzi* vaccine should be focus on the reduction of chronic inflammation-associated damage, Traspain+c-di-AMP proved to be the best strategy assayed in terms of vaccine effectiveness conferring protection against diferent strains of parasites and mice independently of the sex bias of *T. cruzi* infection.

Considering that antigen load is an essential factor for the priming of T cell responses,^30^ it is clear that Traspain allows a lower amounts of antigen (10 µg of Transpain contains a lower amount of molecules than 10 µg of each antigen alone) to be presented more efficiently when we compared the triggered immune response to that obtained with each antigen alone. Moreover, when the molar dose of each antigen was reduced to that of Traspain in Nt-Cz+ASP2+c-di-AMP group, the immune respose although from the same quality was not as robust and failed to achieve similar levels of protection (Fig. 6c). In addition, since the chimeric molecule presents higher MW, pI, number and distribution of charged residues, the physicochemical differences of each formulation could influence self-assembly processes including particle or aggregates formation increasing the probability of protein uptake and antigen processing. Electron microscopy, analitycal SEC and DLS assays should reveal this issues in futher studies. Furthermore, we could not discard an scenario, where DC competition for the antigen uptake and processing takes place as well as the possibility that one antigen may interfere with the other during steps of T cell priming in Nt-Cz+ASP2+c-di-AMP group.

This is the first report to present a chimeric molecule containing the Nt-Cz and ASP-2, both main *T. cruzi* vaccine candidates, which showed protection during the whole course of the infection. Despite the immune response demonstrated towards the linker motive, its protective quality alone remains to be proved in further studies. The intrinsic complexity of *T. cruzi*, with more than 12,000 protein coding genes per haploid genome, six discrete type units, and the presence of protein superfamilies with repetitive and polymorphic variants^31, 32^ highlights the difficulty in finding only one target that confers acceptable levels of protection against the infection. Designing an anti-*T. cruzi* vaccine is challenging not only because of these facts but also due to the kind of immune response required in order to achieve protection. Thus, cell-mediated immunity, particularly CD8^+^ T cells, plays a crucial role in controlling parasite infection.^2^ Considering this scenario, we believe that a multicomponent vaccine is appropriate in order to increase the breadth of the immune response triggered by vaccination. Our results highlight the importance of designing chimeric molecules in multicomponent vaccines a fact that must be considered not only to reduce production costs but also to seek for an improvement in the magnitude and quality of the immune response elicited by each component.

Prompt control of *T. cruzi* infection might require the identification of early antigens such as flagellar proteins^33^ and the incorporation to new chimeric molecules as Traspain represent an attractive area of research for the development of novel anti-*T. cruzi* vaccines.

## Materials and methods

### Mice and parasites

Female C3H/HeN (H-2k) mice 6–8-weeks-old were kept at the animal facility of the Helmholtz Center for Infection Research under specific pathogen-free (spf) conditions. C57BL/6 (H-2b) mice were maintained in the University of Georgia animal facility under spf conditions. Animal experiments were approved by the ethical board and conducted in accordance to the regulations of Lower Saxony No. 09.4250204105/07, Germany, UBA-CONICET, Argentina and IACUC, UGA, USA. *T. cruzi* bloodstream trypomastigotes of the RA and the recombinant Tulahuen strain expressing *β*-galactosidase^34^ were isolated from infected mice. Tissue culture trypomastigotes of the CL strain of *T. cruzi* wild-type or expressing tdTomato were obtained from passage through Vero cells.^35^

### Recombinant proteins

The Nt-terminal domain of Cz (Nt-Cz) was produced as previously described.^14^ The ASP2 transcript was obtained from *T. cruzi* amastigote cDNA from the RA strain as reported.^36^ The central region of ASP2 protein (residues 261–570), herein referred to as ASP2, was amplified employing gene-specific primers and cloned in a pET23a vector. For Traspain construction, sequential PCR was done to build the alpha helix linker incorporating nucleotides 1205–1281 from CL-Brener iTS (XM_811430.1) to Nt-Cz. Splicing by overlap extension PCR^37, 38^ was performed in order to obtain the hybrid gene with ASP2. Cloning was done in a pET23a vector. Gene sequencing was performed to confirm the chimeric gene. Traspain and ASP2 were expressed in *E. coli* BL21 (DE3) as inclusion bodies, purified under denaturing conditions by IMAC and in vitro refolded by dialysis method. Purity levels were determined by SDS-PAGE and presence of endotoxin was assessed by LPS-Detection Kit (invivogen).

### Immunizations and challenge

Inbred female C3H/HeN mice 6–8-weeks-old were immunized by the intranasal route with three doses every 15 days as follows: (I) c-di-AMP, (II) Traspain, (III) Traspain+c-di-AMP, (IV) Nt-Cz+ASP2+c-di-AMP, (V) ASP2+c-di-AMP, and (VI) Nt-Cz+c-di-AMP. Each group received 10 µg of each component, with the exception of group IV that received equal molar amounts of each antigen. For analysis of the acute phase, 15–30 days after the last dose, mice were challenged with 10^3^ *T. cruzi* RA strain blood trypomastigotes by the intraperitoneal route. For sublethal assays 10^2^ parasites were administered. Alternatively, male C57BL/6 were immunized following the same schedule and challenged in the hind footpads with 2.5 × 10^5^ *T. cruzi* tdTomato trypomastigotes.

### ELISA titration of antigen-specific IgG Abs

Protein-specific antibody titers were determined as previously described^39^ by ELISA using plates coated with 0.2 μg of Traspain, Nt-Cz, or ASP2 per well. Peroxidase conjugated goat immunoglobulins to mouse immunoglobulin (Ig) G were used as the secondary antibody. Plates were developed by adding o-phenylenediamine/H_2_O_2_. For peptide-ELISA, plates were coated with 10 µM of GELRIIKSV in carbonate buffer pH 9.6 ON, washed and blocked, sera was incubated ON. Biotin conjugated goat immunoglobulins to mouse IgG plus streptavidin-peroxidase were used as the secondary antibody. Plates were developed by adding TMB/H_2_O_2_.

### Neutralization assay

Raw cells (5 × 10^3^ cells/well) were infected with blood trypomastigotes expressing *β*-galactosidase at a MOI of 10:1 for 24 h at 37 °C. Trypomastigotes were pre-incubated in triplicate with diluted serum (1/10) from mice belonging to each immunization group. After overnight incubation cells were washed and incubated for 5 days. CPRG was added to determine the levels of parasites as previously described.^40^

### Proliferation assay

Spleen cells (5 × 10^5^ cells/well) of each vaccination group were incubated in quadruplicates for 96 h in the presence of different concentrations of Traspain (1, 10, 20, and 40 µg/ml) and proceeded as previously reported.^20^ Results were expressed as the ratio of mean values from stimulated and non-stimulated samples, proliferation index (PI).

### ELISPOT assays

Spleen cells (4 × 10^5^/2 × 10^5^ cells/well) were incubated for 24 h (IFN-*γ*) or 48 h (IL-2, IL-17, and IL-4), in the absence or presence of 10 µg/ml of either Traspain or ASP2 or Nt-Cz for ELISPOT assays (BD Pharmingen, USA). After incubation, cells were removed and the plates were processed according to the manufacturer’s instructions. Colored spots were counted with an ELISPOT reader (CTL S5 Micro Analyzer) and analyzed using ImmunoSpot image analyzer software v3.2 (CTL Europe GmbH, Germany).

### Intracellular cytokine staining

Isolated splenocytes were stimulated overnight with 10 µg/ml of Traspain in the presence of anti-CD154-PE and anti-CD107 PE-Cy7. Brefeldin A plus monensin was added to cultures during the last 6 h of incubation. Surface staining was performed with anti-CD3e-V500, anti-CD4-APC-H7 (BD), and anti-CD8α-Brilliant-Violet-650 (BioLegend). Cells were fixed with PFA 2%, permeabilized in 0.5% saponin, and stained using anti-IFN-*γ*-Brilliant-Violet-711 (BioLegend) and anti-TNF-*α*-eFluor450 (eBioscience) in accordance with the manufacturer’s instructions.

### MHC class I multimer staining

To detect antigen-specific T cells, spleen or blood cells were first labeled with H2K^k^-TEWETGQI dextramer-APC (Immudex) and then with anti-CD3e V500, anti-CD4-APC-H7 (BD), and anti-CD8α-Brilliant Violet V650 (BioLegend) according to the manufacturer’s instructions. Alternatively, either H2K^b^-VNHRFTLV-APC or H2K^b^-ANYKFTLV-APC (Tskb20) tetramer (NIH Tetramer Core Facility, Emory University, USA) were employed for C57BL/6 mice.

### In vivo cytotoxicity assay

Splenocytes collected from naïve C3H/HeN mice were incubated with 5 μM of peptides, pep1: TEWETGQI (ASP2), pep2: KEEASSAV (Nt-Cz), and pep3: GELRIIKSV (*α*-linker*-*iTS) or no peptide for 30 min at 37 °C and 30 min at 4 °C, washed, and then labeled with 2.5, 5, 10, and 0.5 μM of CFSE (CellTrace™ CFSE Cell Proliferation Kit), respectively. Cells were washed, combined, and transferred (4 × 10^7^ total cells) intravenously to syngenic naïve (c-di-AMP), traspain+c-di-AMP immunized, and *T. cruzi* infected mice at 45 dpi. Sixteen hours after transfer, spleens were harvested and analyzed by flow-cytometry. The percentage of specific lysis was calculated as follows: % Specific lysis = 1−[(%CFSE_pep_/%CFSE_unloaded_) Immunized/(%CFSE_pep_ /%CFSE_unloaded_)naïve]  * 100

### Assessment of vaccine efficacy

Parasitemia was monitored by counting peripheral parasites every 2 days as previously described.^40^ In tdTomato *T. cruzi* infection the fluorescent intensity was measured using a whole animal imaging system (Maestro2 In Vivo Imaging System CRi, USA) as a surrogate of parasite load.^35^ Muscle injury was evaluated through the determination of a panel of myopathy-linked enzyme markers as previously described.^39^ The histological features of heart and skeletal muscles were also investigated. A blind histological test was performed as previously described.^41^

### Statistical analysis

Statistical analysis was carried out with Graphpad Prism 6.0 software (San Diego, CA, USA) using one-way ANOVA, *n* = 6 animals/group unless otherwise specified in figure legends, *p*-values <0.05 were considered significant.
